# A Review on Recent Contribution of Meshfree Methods to Structure and Fracture Mechanics Applications

**DOI:** 10.1155/2014/247172

**Published:** 2014-01-02

**Authors:** S. D. Daxini, J. M. Prajapati

**Affiliations:** ^1^Department of Mechanical Engineering, Babaria Institute of Technology, Vadodara, Gujarat 391240, India; ^2^Department of Mechanical Engineering, M. S. University, Vadodara, Gujarat 390020, India

## Abstract

Meshfree methods are viewed as next generation computational techniques. With evident limitations of conventional grid based methods, like FEM, in dealing with problems of fracture mechanics, large deformation, and simulation of manufacturing processes, meshfree methods have gained much attention by researchers. A number of meshfree methods have been proposed till now for analyzing complex problems in various fields of engineering. Present work attempts to review recent developments and some earlier applications of well-known meshfree methods like EFG and MLPG to various types of structure mechanics and fracture mechanics applications like bending, buckling, free vibration analysis, sensitivity analysis and topology optimization, single and mixed mode crack problems, fatigue crack growth, and dynamic crack analysis and some typical applications like vibration of cracked structures, thermoelastic crack problems, and failure transition in impact problems. Due to complex nature of meshfree shape functions and evaluation of integrals in domain, meshless methods are computationally expensive as compared to conventional mesh based methods. Some improved versions of original meshfree methods and other techniques suggested by researchers to improve computational efficiency of meshfree methods are also reviewed here.

## 1. Introduction

Numerical simulation has proved to be a good alternative scientific investigation tool to expensive, time consuming, and sometimes dangerous experiments in complex engineering problems. Grid based numerical methods, like FEM, are widely used for analyzing various engineering problems. There are two fundamental approaches in grid based methods: Eulerian and Lagrangian grid. To strengthen the advantages of each approach and avoid their limitations, new combined approaches were also developed [1]. But grid based methods are not well suited to treat the problems of fracture mechanics with moving material discontinuity, large deformation problems where excessive mesh distortion takes place, and when simulation of some manufacturing process is to be studied.

By modifying the internal structure of gird based method, meshfree methods were developed which are expected to be more adaptive, versatile, and robust and can deal with problems where conventional methods are not suitable. The concept of the meshfree methods is to provide accurate and stable numerical solutions for integral equations or PDEs with all types of possible boundary conditions with a set of arbitrarily distributed nodes without defining mesh which connects these nodes [1]. Many meshfree methods have been developed till now. Earliest meshfree method was developed in 1977 by Lucy and Gingold and Monaghan as smoothed particle hydrodynamics (SPH), a meshfree particle method [2–4]. It was initially developed for modeling astrophysical phenomena but later widely used for applications of solid and fluid mechanics. Many corrected versions of SPH were proposed by researches to solve problems of instabilities and inconsistencies in original SPH model. Later, Nayroles et al., in 1992, were the first to use moving least square approximations in a Galerkin method to formulate the so-called diffuse element method (DEM). Based on the DEM, Belytschko et al., in 1994, advanced remarkably and proposed the element free Galerkin (EFG) method, which was the first meshfree method based on global weak form [5]. Atluri and Zhu, in 1998, had originated the meshless local petrov-Galerkin (MLPG) method, based on local weak form that requires only local background cells for the integration [6]. Liu and his coworkers proposed reproducing kernel particle method (RKPM) [7]. Subsequently, other meshfree methods were developed by researchers, like hp cloud method by Duarte and Odent 1996; point interpolation method (PIM) by Liu and Gu, 1999, Wang and Liu, 2000, 2001, 2002, and meshfree weak-strong form (MWS) by Liu and Gu 2002, 2003 [1]. Some of the important features of meshfree methods are as follows, which makes them superior [8]:

- there is no mesh alignment sensitivity, and mesh used in background is for integration purpose;
- node connectivity is not predefined by mesh;
- no remeshing is required especially in case of large deformation and moving discontinuity problems;
- shape functions of any desired order continuity can be constructed;
- no postprocessing required for smooth derivatives of unknowns and their derivatives.

**Figure 1:**
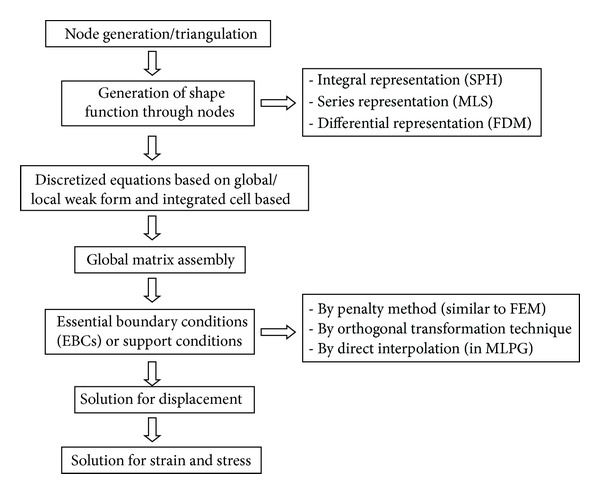
Procedural steps in meshfree methods.

Procedural steps involved in developing solution using meshfree method are shown in Figure 1.

Some review papers have also been presented in the area of development and applications of meshfree methods, earlier. Like, Belytschko et al. presented review on meshless approximation based on MLS, reproducing kernels (RK) and partition of unity methods (PUM) in 1996. The review included the following aspects: salient features of these methods, techniques to handle material and geometric discontinuity, implementation issues, like EBCs, coupling with finite elements, computational efficiency, convergence rate, and so forth, review of applications of plate and shell problems [9]. Li and Liu, in 2002, reviewed recent development of meshfree particle methods and their application in applied mechanics. Major approaches reviewed by them were SPH, meshfree Galerkin methods like DEM, EFG, MLPG, and hp cloud and some applications of molecular dynamics (MD) [10]. Nguyen et al., in 2008, presented a review on meshless methods and its computer implementation aspects with the aim of providing practical overview of meshless methods based on global weak form through a simple and well-structured MATLAB code including intrinsic and extrinsic enrichment, some boundary condition enforcement schemes and few one, and two dimensional numerical examples [11]. Liew et al., in 2011, presented their review on meshless methods for laminated and functionally graded plates and shells, wherein EFG and RKPM methods and their applications, including static and dynamic analysis, buckling, free vibration, and non-linear analysis, were in focus [12].

The present paper attempts to review recent and some earlier applications of some of the well-known meshfree methods like EFG and MLPG, without giving mathematical description, to structure and fracture mechanics problems. The outline of paper is as follows.  Section 2 gives basic concepts of different aspects in meshfree methods like shape functions, weight functions, techniques for imposing essential boundary conditions and numerical integration, and so forth.  Section 3 presents review of EFG and MLPG applications to various structure mechanics problems.  Section 4 presents review of MMs applications to fracture mechanics problems. While review of some typical applications and techniques developed for improving computational efficiency of MMs is presented in Section 5 followed by conclusion and discussion of review in Section 6.

## 2. EFG and MLPG

Meshfree methods are classified based on use of global or local weak form to derive system matrices. Accordingly, EFG method is based on global weak form, while MLPG method is based on local symmetric weak form (LSWF). In both these methods, approximation is based on moving least square (MLS) approximants. But in moving least square methods interpolants do not pass through data point as interpolation functions are not unity at nodes [5]. Hence, imposition of essential boundary conditions (EBCs) gets complicated in these methods. Present section provides basics of shape function construction, selection of weight functions, and techniques to impose essential boundary conditions and integration.

### 2.1. MLS Approximations

The basic idea of MLS approximation is based on construction of set of nodes in the problem domain and hence the method is element free [5].

The MLS approximant *u* ^*h*^(*x*) of the function *u*(*x*) defined over the domain *Ω* is given by

(1) $$\begin{array}{c} u^{h}\left(x\right)=p^{T}\left(x\right)a\left(x\right),\;\forall x\in \Omega , \end{array}$$

where *p* ^*T*^(*x*) are monomial basis functions of order m and *a*(*x*) are vector coefficients which are functions of space coordinates *x*, which can be determined at any point *x* by minimizing weighted discrete *L*_2_ norm defined as follows,

(2) $$\begin{array}{c} J\left(x\right)=\sum_{i=1}^{n}w_{i}\left(x-x_{i}\right){\left[p^{T}\left(x_{i}\right)a\left(x\right)-u_{i}\right]}^{2}, \end{array}$$

where, *n* is the number of nodes in neighborhood of *x* for which weigh function *w*_*i*_ (*x* − *x*_*i*_) cannot be zero and *u*_*i*_ is the nodal value of *u* at *x* = *x*_*i*_. On further solution for *a*(*x*), final expression for MLS approximants is given by

(3) $$\begin{array}{c} u^{h}\left(x\right)=\sum_{i=1}^{n}\Phi_{i}\left(x\right)u_{i}, \end{array}$$

where Φ_*i*_(*x*) is called the shape function of MLS approximation. Detailed shape function construction can be referred from references [5, 13].

### 2.2. Selection of Weight Functions

Weight function selection is also an important parameter while developing the meshfree solution. It should be constructed in such a way that their value should decrease as the distance from *x* to *x*_*i*_ increase. Selected weight functions must be positive and the function and its derivative should be continuous up to required degree [5]. Some of the weight functions used are as the following.

Gaussian weight function:

(4) wi(x)=exp[−(di/ci)2k]−exp[−(ri/ci)2k]1−exp[−(ri/ci)2k],0≤di≤ri=0, di≥ri,

spline weight function:

(5) wi(x)=1−6(diri)2+8(diri)3−3(diri)4, 0≤di≤ri=0, di≥ri,

where *d*_*i*_ = |*x* − *x*_*i*_| is the distance from node *x*_*i*_ to any point *x*, *c*_*i*_ is the constant controlling shape of the weight function, and *r*_*i*_ is the size of the support. There are several other types of weight functions used like conical weight function, cubic spline weight function, and so forth.

### 2.3. Imposing Essential Boundary Conditions

Because MLS shape functions used in EFG and MLPG do not satisfy Kronecker delta criterion, process of imposition of EBCs gets complicated than FEM. Number of techniques were developed for enforcing EBCs in the problem like Lagrange multiplier, penalty method, orthogonal transformation techniques, coupling with FEM, Nitsche's method, singular weighing functions, boundary collocation, and D'Alembert's Principle. Out of these techniques, penalty method can be easily implemented and do not increase much computational effort. Detailed description of these techniques can be referred from references [5, 14–19].

### 2.4. Integration Techniques

Several different techniques were suggested for numerical integration of Galerkin weak form. Gauss quadrature is most commonly employed technique to evaluate integrals in Galerkin weak form. Integration in meshfree method is based on background cells which are independent of nodes. Background cells serve important purpose of identifying nodes contributing to discrete *L*_2_ norm at a quadrature point [8]. By minimizing the mismatch of shape function local support domain with integration cells, integration errors can be minimized and accuracy and convergence can be improved [20]. But because of some inherent drawbacks like complexity, requirement of higher order quadrature rules, specialized integration zone patterns, and so forth, direct nodal integration technique was proposed. But it led to oscillations in solution due to under-integration of weak form and vanishing shape functions at nodes. To alleviate this issue, a stabilized nodal integration technique was proposed by adding a residual of the equilibrium equation to the potential energy function which does not need background cell structure and results in completely meshless method [21, 22].

## 3. EFG and MLPG: Structure Mechanics

Meshless methods developed, in their original form, are not entirely “meshless” and each method falls in one of the following categories: methods based on global weak form requiring background cells for integration like EFG, methods based on local weak form requiring background cells locally like MLPG, and particle methods which require predefinition of particles for their volume or mass like SPH [8]. Following sections review applications of EFG and MLPG to structure and fracture mechanics problems.

### 3.1. Static and Dynamic Analysis

The element-free Galerkin (EFG) method was developed by Belytschko et al. based on the diffuse elements method (DEM) originated by Nayroles et al. In original form of EFG, moving least square (MLS) approximants were used to approximate field variables but due to lack of Kronecker delta property in MLS shape functions, essential boundary conditions (EBCs) cannot be imposed in straight forward way as in FEM and some special techniques are required. Lagrange multiplier technique was used for enforcing EBCs. Because EFG method is based on global weak form, it requires a mesh of background cells for integration in computing the system matrices. Proposed EFG method had advantages like high rate of convergence, no post processing for unknowns or their derivatives, and suitability to fracture mechanics problems [5]. Several techniques for enforcing EBCs in meshfree Galerkin method were proposed. The same authors proposed modified variational principle instead of Lagrange multiplier for imposing EBCs in EFG method [14]. A new technique for imposing EBCs in meshfree methods was proposed by Krongauz and Belytschko, wherein finite elements were used along the essential boundaries and shape functions of finite elements were combined with approximants used. High rate of convergence was observed with implementation of present technique [15]. Another boundary condition enforcement technique was proposed by Günther and Liu by a computationally efficient algorithm based on D'Alembert's principle that can be used for general constraints and fluid structure interface in meshless methods [16]. MLS shape functions used in EFG method are more complex than piecewise polynomial like shape functions used in FEM; hence Krysl and Belytschko presented a straightforward way to program the EFG shape function construction in a way which leads to both a simple interface to application code and to the implementation of EFG shape function itself [23]. Dolbow and Belytschko proposed EFG method implementation with its structured MATLAB code to benchmark structure problems in one-dimensional and two-dimensional applications. To enforce EBCs, few techniques were suggested like Lagrange multiplier, modified variational principles, and coupling with finite elements [24]. To solve three-dimensional elastic and elastoplastic problems, EFG method was proposed by Barry and Saigal with variable domain of influence approach. Singular weight functions were utilized in MLS shape functions allowing accurate and direct nodal imposition of EBCs. Several elastic and small strain elastoplastic problems were presented [25]. Tiago and Leitao applied EFG method to free vibration analysis of beams and plates. Shape functions were constructed by MLS approximation and kinematic boundary conditions were imposed by Lagrange multiplier technique in their work [26]. To improve computational efficiency of original EFG method, Zhang et al. presented improved EFG (IEFG) method by employing improved MLS (IMLS) shape functions for two-dimensional potential problems. MLS approximants yield precise solution but sometimes final algebra equations are ill-conditioned, which is undesirable. Improved MLS (IMLS) approximation was proposed by Liew et al. to alleviate the problem in boundary element method [6]. Proposed improved EFG method uses weighted orthogonal basis function for construction of MLS shape functions which avoids ill-conditioned algebra equations as in case of conventional MLS interpolation. IEFG method uses fewer nodes in entire domain than conventional EFG method and resulting in higher computation speed [27]. Proposed IEFG approach was extended for solving three-dimensional potential problems by same authors [28]. In a more recent development, for two dimensional elastoplasticity problems, complex variable moving least square approximation (CVMLS) and EFG based CVEFG were proposed by Peng et al. With CVMLS, it becomes possible to select fewer nodes in the meshless method than are required in the meshless method of the MLS approximation without loss of precision or in other words, CVMLS is computationally more efficient [29]. Presently, EFG method is one of the most popular meshfree methods, and applied to many structure and fracture problems, some of which are reviewed here in subsequent sections.

Another new meshfree computational method was developed and proposed by Atluri and Zhu, known as meshless Local Petrov Galerkin method (MLPG), based on local weak form and MLS approximants. EBCs are imposed by penalty method in MLPG. Selection of trial (shape) function and test function in MLPG is done from entirely different spaces and it is considered as a truly meshless method because all integrals can be easily evaluated over regular shaped domains and their boundaries. High convergence rate and accurate values of unknown variables and its derivatives were observed [13]. Elastostatic problems, like an infinite plate with circular and elliptical hole, were addressed by the same authors using MLPG method [30]. MLPG is a general concept; hence a comparison study of the efficiency and accuracy of a variety of meshless trial and test functions for different variants of MLPG was proposed by Atluri and Shen, wherein five types of trial functions and six types of test functions were explored and six different approaches, popularly known as MLPG1 to MLPG6, were presented. Numerical results for standard patch test, Laplace and Poisssion's equations, were compared for efficiency and computational cost and MLPG5 was found less expensive from computational view point as it employs local, nodal based test function over a local subdomain, a Heaviside step function [31]. Long and Atluri proposed MLPG for bending of thin (Kirchhoff) plates based on MLS approximants and local symmetric weak form (LSWF). Cubic, quartic, and quintic basis, as well as the quitic spline weight function was employed in the MLS computation, while EBCs were enforced by penalty method in proposed computation [32]. Raju and Phillips had shown MLPG application for Euler Bernoulli beam problems like cantilever beam and simply supported beam with various loading conditions by selecting simple weight functions as test functions and MLS approximation [33]. The same authors presented MLPG method for Euler-Bernoulli beams with radial basis function as trail function instead of GMLS interpolation functions and test functions as simple weight function. Radial basis interpolation function yields computationally simple method involving fewer matrix inversions and multiplications. Effectiveness of proposed MLPG method was evaluated by the number of patch test and mixed boundary value problems [34]. Li et al. extended MLPG approach to three-dimensional elastostatic problems by combining two methods of MLPG family, MLPG2 and MLPG5, in order to achieve high computational efficiency. MLPG5 was applied to domain from inside to eliminate domain integration and MLPG2 was applied at nodes on boundaries and interfaces of material discontinuities so that boundary conditions and material discontinuities are satisfied. Results obtained by application of proposed technique have shown good agreement with analytical solution [35]. Han and Atluri developed MLPG method for solving three-dimensional elastodynamic problems which was derived from LSWF of the equilibrium equations by general MLPG concept and MLS shape functions. The present numerical technique imposes a correction to the accelerations to enforce the kinematic boundary conditions in MLS approximation with explicit time-integration algorithm [36]. While Long et al. proposed a new MLPG approach based on MLPG5 and coupled radial basis function (RBF) with polynomial basis function as trial function for elastodynamic problems. The shape function constructed possesses Kronecker delta property, hence no additional treatment to impose EBCs are required. Newmark family of methods is adopted in time integration scheme and the technique does not involve any domain or singular integration [37]. As a new concept, MLPG approach with polygonal sub domains constructed from several triangular patches rather than typically used circular subdomains was presented by Pudjisuryadi. Variable domain of influence (VDOI) and effective stress gradient indicator for assessing local errors were focused in the study. VDOI helps in alleviating the problem raised by adaptive meshfree approach where problem domain is refined with new nodes placed in area where local error exceeds a level but due to constant size of influence domain, node density in that area goes too high and finally it leads to higher computational cost and ineffective adaptive technique [38]. In a recent development, a novel MLPG method with new test function, Guassian test function, as a schema to solve problems in elastostatic and fracture mechanics was developed by Abdollahifar et al. Four different variants of MLPG method, MLPG1, MLPG2, MLPG5, and MLPG6, can be approached using new test function and sufficiently accurate results were obtained [39].

### 3.2. Plates and Shell Problems

Due to the flexibility in constructing approximation functions with desired smoothness and accuracy in meshfree methods, they have been successfully applied to Kirchhoff type of plates and shell problems. Applications to plates were first investigated by Hein [40] but due to use of point collocation to enforce EBCs, too small supports and unsuitable weight functions desired results were not observed. Lu et al. [41] treated Mindlin-Reissner plates with linear and quadratic basis EFG method, but results were poor due to shear locking. The EFG method had been applied to thin (Kirchhoff) plates by Krysl and Belytschko, in 1995. Background quadrilateral elements were used for the purpose of Gaussian numerical integration. An attempt to optimize the accuracy of the method by the choice of the weight function support size was undertaken [42]. Same authors applied EFG method to thin shells wherein background elements were used for surface shape approximation and numerical integration. MLS was used in approximating, surface while EBCs were imposed by Lagrange multiplier. To achieve consistency, quadratic and quartic polynomial basis was used along with quartic spline weight function [43]. Liu and Chen applied EFG method with MLS approximation to static and free vibration analysis of thin plates of complicated shapes, that is, rectangular plate, elliptical plate, and complicated shapes with different boundary conditions. In proposed approach, for static analysis EBCs were imposed by penalty method while in free vibration analysis they were imposed by orthogonal transformation technique [44]. Free vibration analysis of composite laminates of complicated shapes through EFG method was carried out by the same authors. EBCs were imposed by Lagrange multiplier and orthogonal transformation technique. Numerical examples of square plate, elliptical plate, and other complicated shapes were addressed by proposed method [45]. Dai et al. also presented a meshfree method for analyzing thin and thick laminated composite plates for static deflection and natural frequencies using higher order shear deformation theory. MLS approximants were applied to construct the shape functions and variational principle was used to derive the discrete system equations based on the third order shear deformation theory (TSDT) of Reddy. EBCs were enforced by a penalty technique for both the static deflection and natural frequency analysis [46]. The same authors presented EFG method for thermomechanical analysis of FGM plates containing distributed piezoelectric sensors and actuators with structured and unstructured (irregular) node arrangement. The weak form was formulated based on FSDT and shape functions were constructed by MLS functions. EBCs were imposed by penalty method in proposed Galerkin method. Unstructured nodes were also giving the desired order of accuracy in results [47]. Peng et al. proposed EFG method for static analysis of concentrically and eccentrically stiffened plates based on FSDT. The influences of support size and order of the complete basis function on the numerical accuracy were also investigated and it was observed that larger support size and higher order of basis function will furnish better convergence results [48]. Corrugated plates are widely used in industries due to their improved strength to weight ratio. They can be modeled and analyzed either by considering them as shells or orthotropic plates. For elastic buckling analysis of stiffened and unstiffened corrugated plates with FSDT, meshfree Galerkin method was proposed by the same authors. Corrugated plates were treated as orthotropic plates and stiffeners were taken as beams. Stiffness matrix for structure was obtained by superimposing the strain energy of the orthotropic plate and the beams and imposing the displacement compatibility conditions between the plate and the beams [49]. For analyzing elastic bending of stiffened and unstiffened corrugated plates, meshfree Galerkin method was proposed by the same authors where MLS shape functions with full transformation method was employed for enforcing EBCs [50]. The proposed method was extended for solving nonlinear problems of stiffened and unstiffened corrugated plates based on FSDT and von Karman large deformation theory. For validation of proposed approach, different corrugated plates were analyzed and results were compared with results obtained with shell elements in ANSYS [51]. Belinha and Dinis also proposed EFG method for nonlinear analysis of plates and laminates with FSDT and MLS approximants as shape functions. Laminate bending problems were solved and results were compared with FEM solution [52]. While two members of MLPG family, MLPG1 and MLPG5, were used for three-dimensional static analysis of thick functionally graded plates by Vaghefi et al. wherein MLPG1 uses fourth order spline function as test function and MLPG5 uses Heaviside step function as test function. Young's modulus is considered to be graded through the thickness of plates by an exponential function while Poission's ration is taken as constant while LSWF was derived. 3D MLS approximation was used for field variables and brick shaped domains were considered as local subdomains and support domains [53]. Mojdehi et al. also proposed MLPG for three-dimensional (3D) static and dynamic analysis of thick functionally graded plates using three-dimensional MLS shape function and Heaviside step function as test function [54]. In order to estimate maximum sustainable load by any structure, limit analysis proved to be useful technique where fundamental theorems of plastic analysis are used. For such an application *h*-Adaptive EFG method with MLS approximation was proposed by Le et al. for limit analysis of plates. Accuracy of limit analysis is often affected and decided by local singularities arising from localized plastic deformations and to capture it accurately automatic h-refinement is performed. Taylor expansion technique is used for error estimation in computed displacement field to identify the area needing refinement [55]. In another steel structural application, use of beams with irregular web holes of different shape and arrangement is widespread. But the behavior of such beams is complicated due to irregularity of openings and it needs more reliable technique for their local buckling response. Abidin and Izzuddin proposed EFG method for local buckling analysis of steel beams with irregular web openings. The proposed EFG approach was based on general formulation of plate buckling, where singularity in tangent stiffness matrix was made up of material stiffness matrix and geometric stiffness matrix. MLS approximation was used for shape function construction and the proposed approach was applied to three-dimensional beam buckling problems [56]. In a recent development, Jaberzadeh et al. developed EFG method for buckling analysis of inelastic skew plates with or without line supports. The governing differential equation for a plate in plastic range of response is numerically solved with Galerkin method and Stowell theory for the plastic buckling of flat skew plates with variable thickness is used. MLS approximants are used for shape function construction and EBCs are imposed by Lagrange multiplier, nd orthogonal transformation techniques in proposed method [57]. Spatial thin shell structures are used extensively in many engineering structures including aircrafts, pressure vessels, and automobiles due to its outstanding efficiency in material utilization. Liu et al. applied EFG to thin shell structures for static deformation and free vibration analysis. MLS was used for construction of shape functions and surface approximation of general spatial shell geometry and discrete system equations were obtained by incorporating these interpolations into the Galerkin weak form. EBCs were imposed by penalty approach, Lagrange multiplier, and orthogonal transformation techniques [58]. A meshfree method for static analysis of FGM cylinders was presented by Foroutan et al., wherein mechanical properties were assumed to vary in radial direction. EBCs were imposed by transformation method and MLS approximants were used for approximating unknown filed variable. The method was applied to finite and infinite length cylinders and results obtained were in good agreement with FEM [59].

### 3.3. Large Deformation and Contact Problems

Numerical simulation of contact between two different objects like sheet metal forming, vehicle crash worthiness, impact, penetration, and so forth, is a challenging task. While dealing with contact problems, mesh density must be maintained at a sufficiently high level around contact region to obtain reasonably accurate results. Li et al. proposed a contact detection algorithm based on moment matrix of meshfree approximation. The mathematical principle of contact detection algorithm is that the determinant of moment matrix can automatically determine Lagrangian movement of continuum and on the basis of that one can accurately detect contact or penetration without solving any complex equations. It was implemented to simulate Taylor bar impact problem which is a deformable solid bar impacting rigid target problem [60]. Large strain problems, like hyperelastic materials undergoing large deformations, cannot be handled with ease in FEM due to excessive mesh distortion, but meshfree methods proved to be a good alternative in those cases. Tiago and Pimenta implemented EFG with MLS approximant to nonlinear analysis of plates undergoing arbitrary large deformations which is based on a unified nonlinear theory of plates allowing arbitrarily large rotations and displacements. Presented approach was hybrid in nature where solution was obtained by the independent approximation of the generalized internal displacement fields and generalized boundary tractions [61]. Hu et al. developed MLPG approach for large deformation contact analysis of elastomers like rubber block and compression of rubber ring. Proposed MLPG approach was based on a local weak form with RBF coupled with polynomial basis function. In the present technique, two different sets of equations were used for nodes on the contact surface and nodes away from contact surface [62]. Li and Lee developed an adaptive meshless method, with sliding line algorithm and penalty method to handle contact constraints, for solving contact problems involving large deformation in which additional nodes are added automatically into large error regions. For automatic node insertion, a modified error estimation (built on two different support sizes of a basis function) was proposed to identify regions of large computational errors [63]. A novel complex variable EFG method, improved complex variable EFG, for two-dimensional large deformation problem was developed by Li et al. Based on complex variable theory and moving least-squares (MLS) approximation, the improved complex variable moving least-squares (ICVMLS) approximation was developed. Proposed technique was based on Galerkin weak form, while the penalty method was used to impose EBCs [64]. A recent development in nonlinear solid mechanics is proposed by Ullah and Augarde by developing adaptive meshless approach based on EFG method. An existing error estimation procedure for linear elastostatic problems is extended for nonlinear problems including finite deformation and elastoplasticity. Proposed max-ent EFG method can handle material and geometrically nonlinear problems in solid mechanics including a robust means of transferring data between discretizations [65].

### 3.4. Sensitivity Analysis and Shape Optimization

Design sensitivity analysis and shape optimization is applied to observe and find the variation of response measure like displacement, stress due to variation of some design parameter that is, geometric parameter, and finding optimal layout of a structure within a specified region. Bobaru and Mukherjee demonstrated application of EFG to shape design sensitivity analysis and shape optimization for 2D elasticity problems wherein EFG was used first time with continuous formulation using material derivative approach. Penalty approach was used for imposing EBCs and a numerical example of shape optimization of fillet was used to demonstrate robustness and ability of EFG method. The presented approach can be extended to 3D and nonlinear problems. Another application of EFG to shape optimization in linear thermoelasticity problem was also demonstrated by the same authors [66, 67]. Zhang et al. proposed meshless computational strategies for shape optimal design through the composition of behavioral fields quite similar to Boolean operations in constructive solid geometry (CSG). A meshless approximation using nonuniform rational B-spline basis functions was used to discretize the behavioral fields defined over the geometrical primitives while remeshing was performed for only those primitives that were modified. Due to a tighter integration between design and analysis, it is termed constructive solid analysis (CSA) [68]. Juan et al. introduced a technique to combine EFG with evolutionary structural optimization (ESO) for topology optimization of the continuum structures where objective function of the model is to minimize weight by gradually removing the inefficient material from the design domain. Feasibility and efficiency of the proposed technique was illustrated with several 2D examples like cantilever beams and simply supported beams [69].

## 4. Fracture Mechanics

### 4.1. Static and Dynamic Fracture

Belytschko et al. developed EFG method for linear elastic fracture problems in 1994 where “Visibility criterion” was proposed firstly to model geometric discontinuity (crack) in which domain of influence for nodes near the crack are truncated whenever they intersect the crack surface and hence a node on one side of crack will not affect the point on other side of crack. But “visibility criterion” had difficulty in treating nodes near crack tip. Other improved techniques for handling discontinuity were also suggested: diffraction method, transparency method, and “see through” method or continuous line criterion. Two methods for enriching EFG approximations for linear elastic fracture problems were also proposed by Belytschko: extrinsic and intrinsic enrichment [70, 71]. Belytschko and Tabbara proposed EFG approach for dynamic fracture problem in 1996 involving numerical examples of crack propagation at constant velocity and constant value of dynamic fracture toughness [72], while for dynamic propagation of arbitrary three-dimensional cracks, EFG approach was developed by Krysl and Belytschko. MLS shape function and truncated Gaussian weight functions were employed in proposed EFG approach [73]. Belytschko et al. studied mixed mode dynamic crack growth in concrete using EFG methods wherein fracture process zone (FPZ) technique was used in formulation as linear elastic fracture mechanics (LEFM) is not applicable to concrete and other cement-based materials. Numerical examples of mode I and mixed mode cracks in concrete were discussed [74]. Rao and Rahman proposed an efficient meshfree method, based on EFG, for linear elastic crack problems with single and mixed mode loading conditions. Proposed technique involves a new weight function and exact implementation of EBCs. Estimated SIFs and neat tip stress fields were in good agreement with FEM results and experimental values [75]. Tiago and Leitão also applied EFG to damage analysis of reinforced concrete beams which demonstrated handling material inhomogeneities, discontinuity in geometry, and concentrated loads [76]. Lee and Yoon proposed an enhanced EFG method with enhancement functions to improve solution efficiency for linear elastic fracture problems where singularity and discontinuity of crack were modeled with enhancement function and discontinuity functions, respectively. EBCs were imposed by penalty method and coupling with FEM [77]. Rabczuk and Belytschko proposed a new EFG approach, called EFG-Particle (EFG-P), for modeling discrete cracks wherein crack growth was represented by activation of crack surfaces at individual particles and hence crack's topology representations is not needed. The crack was modeled by local enrichment of trial and test functions with sign function and it can handle crack branching and fragmentation also [78]. One of the most efficient MLPG variant, MLPG5, was used for analysis of elastodynamic deformations near crack tip by Kaiyuan et al. Newmark family of methods was applied into the time integration scheme. A numerical example of a rectangular plate with a parallel central crack loaded in tension was approached by proposed technique [79]. EFG method was extended to solve three-dimensional elastic fracture mechanics problems, mode I and mode II cracks, by Brighenti. Out of different ways for modeling geometric discontinuity by EFG method, “visibility criterion” was used to detect them in present approach, while Gauss type weight function along with penalty approach employed to enforce the boundary conditions. Presented approach was validated by solving thick plate with an edge crack under tension and finite thin plate with central slant crack under tension [80]. Improved element free galerkin method (IEFG) with an improved moving least square approximation (IMLS) was developed for analyzing two-dimensional fracture mechanics problems by Zhang et al. Major advantage with IMLS is its greater computational efficiency than MLS and it does not lead to ill-conditioned system of equations as MLS does sometimes [81]. The effective and accurate calculation of stress intensity factors (SIF) is one of the basic problems in LEFM. Two principal approaches are known for a SIF calculation: local, based on the use of displacements or tractions near to the crack tip; and global or energy methods, based on the calculation of the energy release rate in terms of crack growing. Parvanova presented a procedure for calculation of SIFs based on standard appearance of force-displacement curve using EFG method. The method was used to develop a new idea based on standard appearance of the force-displacement curve in LEFM related to the accurate derivation of the SIF for pure opening mode I type fracture. A MATLAB code for two-dimensional elasticity problems had been worked out, along with intrinsic basis enrichment for precise modeling of the singular stress field around the crack tip [82]. Zhang and Chen proposed a simplified meshfree method with Kronecker delta property for incorporation of displacement boundary conditions for dynamic crack growth problem wherein the crack was presented by a set of rotated crack segments that pass through the entire domain of influence of the meshfree nodes. Rankine criterion was used to initiate the crack and discontinuous displacement field was obtained by an extrinsic enrichment based on a local partition of unity concept [83]. A meshfree analysis of dynamic fracture in thin walled structures was proposed by Gato, where fracture of the shell is modeled by breaking links between particles once a certain fracture criterion is met. For validating the proposed approach, numerical examples of quasistatic tearing of a square plate, an impact problem and detonation driven fracture of cylindrical shells were considered. In present work, it was the first time that the 3D continuum approach based on Lagrangian kernels was applied to fracture of thin shells and implementation was done in C++ [84, 85]. In a recent development, a new enrichment criterion for modeling kinked cracks using EFG method is proposed by Pant et al. In order to capture crack tip stress singularity, some additional terms are incorporated in the linear basis function. The proposed criterion is applied for simulating the quasistatic crack growth in two-dimensional domain subjected to mixed mode loading [86].

### 4.2. Composite Solids

Functionally graded materials (FGM) possesses continuously varying microstructure and material properties in a predetermined way and they are used in structures subjected to nonuniform service conditions. Rao and Rahman proposed EFG approach for calculating stress intensity factors (SIF) for stationary crack in twodimensional functionally graded materials of arbitrary geometry. In proposed method, the interaction integral method was extended for FGM and material properties were taken as smooth functions of spatial coordinates and two newly developed interaction integrals were introduced for analysis of basic modes and mixed mode fracture problems [87]. While two-dimensional stress analysis problems of anisotropic and linear elastic/viscoelastic solids with continuously varying material properties were addressed by Sladek et al., using MLPG with unit step function as test function in local weak form which leads to local boundary integral equations (LBIEs). MLS was adopted for approximating physical quantities in LBIEs and for time-dependent problems, Laplace transformation was utilized [88]. Delamination and matrix cracking are routine damage mechanisms observed during analysis of laminated structures. Guiamatsia et al. proposed EFG for the first time to simulate delamination (interlaminar) and intralaminar matrix microcracking in composite laminates. Modeling was done at mesolevel, where each ply represented individually and background integration cells were arranged per layer in a way that they are not traversed by material interfaces. Virtual crack closure technique (VCCT) was used for crack advancement in the present technique [89]. Orthotropic composites possess high specific strength and stiffness characteristics because of their constituents and extensively applied in various engineering applications. Ghorashi et al. presented a new approach for modeling discrete cracks in two-dimensional orthotropic media by EFG method. In proposed approach, recently developed orthotropic enrichment functions were used which were used earlier in the extended finite element method along with a subtriangle technique for enhancing the Gauss quadrature accuracy near the crack [90].

## 5. Some Typical Applications and Enhancement of Computational Efficiency

### 5.1. Typical Applications

Over a period of time in service, mechanical systems and structures accumulate cracks due to fatigue. Duflot and Nguyen-Dang proposed an enriched meshless method to analyze fatigue crack growth under cyclic loading. The crack propagation was modeled by successive linear extensions determined by SIFs obtained after each linear elastic analysis. A fixed set of three nodes with special weight function were added at each crack tip to accurately catch the stress singularities in proposed approach [91]. Use of modal characteristics, that is, vibration data, like natural frequency and mode shapes, of structures for detecting and predicting cracks has become a good alternative approach because cracked structure's modal data will be different. In order to use vibration data for detecting cracks in load carrying systems or structures, two different theoretical modeling techniques are used: lumped flexibility models and continuous models. Andreaus et al. proposed MLPG approach with MLS shape function for analyzing vibration of beams with multiple cracks. Lumped flexibility model was adopted and each fatigue crack was modeled as rotational spring in proposed approach [92]. In impact problems, transition in failure mode can be observed. But most numerical simulation techniques focus either on brittle failure or ductile failure. Kalthoff and Winkler conducted experiments on prenotched specimen of steel plates, subjected to impact loading with different impact velocities and observed transition in failure modes. Wang and Liu proposed EFG method for simulating failure transition from brittle to ductile under finite deformation. Johnson-Cook damage model was incorporated in Galerkin formulation and EBCs were enforced by collocation method. Node splitting algorithm was used in modeling crack which simplifies the implementation. Proposed method captured the complicated failure transition phenomenon accurately [93]. Under combined mechanical and thermal loadings, the presence of cracks induces a strong variation in fields, which can affect the crack growth direction. While designing structures for turbines, combustion chambers, and nuclear pressure vessels, thermoelastic fracture mechanics aspects need to be considered. Pant et al. proposed intrinsic enriched EFG to solve thermoelastic fracture mechanics problem in homogeneous and inhomogeneous materials (bi-material). The thermo-elastic fracture problem was solved by decoupling it into two separate problems and both the problems were enriched intrinsically to represent discontinuous temperature, heat flux, displacements, and traction across crack surfaces. For modeling bi-material interface, jump function technique had been employed in proposed method [94]. Extended finite element method (XFEM) was developed to ease difficulties in solving problems with geometric discontinuity like cracks by adding discontinuous basis function to standard polynomial basis functions for nodes that belonged to elements intersected by crack. In a recent development, advantages of a meshfree method—EFG are combined with XFEM and extended element free Galerkin method (XEFG) is proposed to model crack propagation under thermomechanical loading by Bouhala et al.. In proposed method, direction of the crack growth is determined by initially calculating SIFs using the interaction energy integral, and then the crack is assumed to propagate in the direction of the maximum principal stress. Shape functions are constructed using MLS approximation and cracks; interfaces and crack tips are modeled with extrinsic local enrichment [95].

### 5.2. Enhancing Computational Efficiency and Error Control

Meshfree shape functions are not interpolation functions and do not possess Kronecker delta properties. Hence imposition of EBCs consumes much computation time. Several techniques were presented for imposing EBCs, like Lagrange multiplier, penalty method, orthogonal transformation techniques, coupling with FEM, Nitsche's method, singular weighing functions, boundary collocation, and D'Alembert's Principle. An overview of existing techniques for enforcing EBCs was presented by Fernández-Méndez and Huerta. With focus on meshfree method coupled with finite elements and methods based on modification of Galerkin weak form [17]. Chen and Wang proposed two new boundary condition treatment techniques, the mixed transformation method and the boundary singular kernel method, to enhance the computational efficiency of meshfree methods for contact problems in RKPM framework. The mixed transformation method is a modification of a full transformation method developed previously for meshfree solution of boundary value problems, while the boundary singular kernel method introduces singularities into the kernel functions associated with the restrained nodes [18]. Another set of new boundary treatment techniques were developed by Ren and Liew, namely, node interpolation method (NIM) and direct imposition method (DIM). In NIM, the shape functions associated with EBCs were constructed using node interpolation and then combined with meshfree shape functions, while DIM rearranges the discretized system equations, and directly provides the known values of the essential boundary conditions in the nodal variable vector [19]. Smoothing of the approximating functions at concave boundaries and accelerated calculations of the approximating function in EFG method was proposed by Belytschko et al. In proposed method, shape functions of EFG method were modified and made continuous in domain with concave corners, by simply redefining a parameter governing decay of weight function [96]. In meshfree methods, for numerical integration of Galerkin weak form, Gauss integration method is most commonly used. Dolbow and Belytschko demonstrated and investigated integration aspects in meshfree methods. Authors emphasized on source of integration errors and suggested techniques to minimize them [20]. But a number of disadvantages have been reported in employing Gauss integration, like complexity, requirement of higher order quadrature rules, specialized integration zone patterns, and so forth. Beissel and Belytschko used direct nodal integration to avoid background cells, but it led to oscillations in solution due to underintegration of weak form and vanishing shape functions at nodes. To overcome it, a stabilized nodal integration technique, for EFG, was proposed by adding a residual of the equilibrium equation to the potential energy function. The proposed technique does not need background cell structure and results in completely meshless method [21]. A stabilized conforming nodal integration (SCNI) method for elastoplastic contact analysis of metal forming processes was proposed by Yoon et al. In this approach, strain smoothing stabilization was introduced to eliminate spatial instability in collocation meshfree methods and convergence was obtained by introducing an integration constraint (IC) as a necessary condition for a linear exactness in the mesh-free Galerkin approximation. Implementation of proposed technique in linear problems demonstrated a significant reduction in computational cost with no loss of accuracy and convergent rate compared to the solution obtained by the use of Gauss integration [22, 97]. In an another application of meshfree formulation with SCNI, Wang and Chen proposed locking free meshfree curved beam formulation based on SCNI with Kirchhoff mode reproducing conditions (KRMC). Proposed meshfree approximation was constructed to represent pure bending mode without producing parasitic shear and membrane deformations. Numerical examples of pure bending of clamped-free curved beam, a nearly straight beam with tip load and a pinched ring demonstrated the technique [98]. Khosravifard and Hematiyan presented a technique for evaluation of regular domain integrals without domain discretization wherein a domain integral is transformed into a boundary integral and a 1D integral and then utilized for domain integrals in meshfree methods based on weak form like EFG method. Presented technique results in truly meshless approach with better accuracy and efficiency. It is known as Cartesian transformation method (CTM) which was used earlier for domain integration in boundary element method by Hematiyan computations, and so forth, Chung [99].

Though meshfree methods look quite attractive for solving a special class of problems, there are issues like error estimate and control, integral evaluation, and accuracy. Errors in numerical modeling arise from number of sources like discretization, quality of mathematical model, rounding off operations in computations, and so forth. Chung and Belytschko, in 1998, proposed estimation of local and global error in EFG method where in error estimation was based on difference between the values of projected stress and stress given by EFG method. Effectiveness of proposed error estimator was validated by various one-dimensional and two-dimensional problems [100]. Zhuang et al. investigated discretization error in EFG method. Discretization errors are arising due to not satisfying governing equation and boundary conditions. Conventional procedures for error analysis used in FEM cannot be applied straightly in meshfree approaches. In FEM, it is feasible to uncouple *h* and *p* adaptivity but in EFG method it is not possible because changing the density of nodes both changes the error *e*_*h*_ and also changes the space of the shape functions and hence the error *e*_*p*_. Hence it is difficult to achieve error control and adaptivity in meshfree methods. In proposed approach the discretization error was split into contributions arising from an inadequate number of degrees of freedom *e*_*h*_ and from an inadequate basis *e*_*p*_ [101]. Kim and Atluri proposed a technique for controlling error and improving solution accuracy in MLPG method by adding and arbitrary placing secondary nodes where better resolution is needed in the domain. But the subdomains for the shape functions in the MLS approximation were constructed only from the primary nodes, and the secondary nodes use the same sub-domains. The proposed technique can become very useful in an adaptive approach, because the secondary nodes can be easily added and/or moved without an additional mesh [102].

## 6. Conclusion and Discussions

Objective of the present work is to provide exposure in terms of versatility of meshfree methods in handling different types of engineering problems without detailed mathematical description. Some of the worth notable recent developments include development of extended element free Galerkin (XEFG) method for thermo-mechanical crack propagation, CVEFG using CVMLS for elastoplasticity problems, ICVEFG using ICVMLS for large deformation problems, simulation of failure transition in impact problems by EFG, local buckling analysis of steel plates with irregular openings by EFG, and adaptive EFG and MLPG approaches.

Though MMs have found application in almost all areas of structure and fracture mechanics, still there are challenges in developing computationally efficient algorithms with accurate nodal integration techniques with scalable implementation of EBCs. Improved versions of original MMs are also proposed by many researchers like XEFG, IEFG, CVEFG, ICVEFG, and so forth, while some of the other techniques for improving computational efficiency are reviewed in Section 5. MMs have exemplary approximation, but the computational cost is the issue. To alleviate the problem, coupled numerical methods like FEM-MM have been developed to exploit potential benefits of each method.
